# Phylodynamics and Molecular Evolution of Influenza A Virus Nucleoprotein Genes in Taiwan between 1979 and 2009

**DOI:** 10.1371/journal.pone.0023454

**Published:** 2011-08-12

**Authors:** Jih-Hui Lin, Shu-Chun Chiu, Ju-Chien Cheng, Hui-Wen Chang, Kuang-Liang Hsiao, Yung-Cheng Lin, Ho-Sheng Wu, Marco Salemi, Hsin-Fu Liu

**Affiliations:** 1 Center for Research and Diagnostics, Centers for Disease Control, Taipei, Taiwan; 2 Institute of Bioscience and Biotechnology, National Taiwan Ocean University, Keelung, Taiwan; 3 Institute of Biochemistry and Molecular Biology, National Yang-Ming University, Taipei, Taiwan; 4 Department of Medical Laboratory Science and Biotechnology, China Medical University, Taichung, Taiwan; 5 Department of Pathology, Immunology and Laboratory Medicine, College of Medicine & Emerging Pathogens Institute, University of Florida, Gainesville, Florida, United States of America; 6 Department of Medical Research, Mackay Memorial Hospital, New Taipei City, Taiwan; 7 Institute of Public Health, National Yang-Ming University, Taipei, Taiwan; University of Cambridge, United Kingdom

## Abstract

**Background:**

Many studies concentrate on variation in the hemagglutinin glycoprotein (HA) because of its significance in host immune response, the evolution of this virus is even more complex when other genome segments are considered. Recently, it was found that cytotoxic T lymphocytes (CTL) play an important role in immunity against influenza and most CTL epitopes of human influenza viruses were remarkably conserved. The NP gene has evolved independently in human and avian hosts after 1918 flu pandemic and it has been assigned a putative role as a determinant of host range.

**Methods and Findings:**

Phylodynamic patterns of the genes encoding nucleoprotein (NP) of influenza A viruses isolated from 1979–2009 were analyzed by applying the Bayesian Markov Chain Monte Carlo framework to better understand the evolutionary mechanisms of these Taiwanese isolates. Phylogenetic analysis of the NP gene showed that all available H3 worldwide isolates collected so far were genetically similar and divided into two major clades after the year 2004. We compared the deduced amino acid sequences of the NP sequences from human, avian and swine hosts to investigate the emergence of potential adaptive mutations. Overall, selective pressure on the NP gene of human influenza A viruses appeared to be dominated by purifying selection with a mean d_N_/d_S_ ratio of 0.105. Site-selection analysis of 488 codons, however, also revealed 3 positively selected sites in addition to 139 negatively selected ones.

**Conclusions:**

The demographic history inferred by Bayesian skyline plot showed that the effective number of infections underwent a period of smooth and steady growth from 1998 to 2001, followed by a more recent rise in the rate of spread. Further understanding the correlates of interspecies transmission of influenza A virus genes from other host reservoirs to the human population may help to elucidate the mechanisms of variability among influenza A virus.

## Introduction

Influenza A virus is a rapidly evolving pathogen, characterized by an extreme genetic diversity that results in the generation of many circulating strains during its epidemic spread [1]. This genetic diversity allows the virus to adapt dynamically to its host and environment. Influenza A variation is clearly the result of intricate interactions between evolutionary and ecological pressures [2]. While many studies concentrate on variation in the hemagglutinin glycoprotein (HA) because of its significance in host immune response [1], the evolution of influenza A virus is even more complex when other genome segments are considered. The Nucleoprotein (NP) gene has been chosen for evolutionary studies of influenza A viruses [3], [4], because the results from a number of studies suggested that the NP gene may be involved in determining host range [5], [6], [7].

The rapid evolution of influenza viruses, and RNA virus in general, occurs on a similar timescale as the ecological dynamics affecting its transmission and spread. Coalescent-based inference methods enable population genetic parameters to be estimated directly from gene sequence data under variety scenarios [8]. However, limited information is available on the molecular evolution and population dynamics of influenza A virus in Taiwan prior to the commencement of the monitoring of influenza activity by the Center for Diseases Control, Taiwan in 2000. The epidemiology of the influenza viruses circulating in Asia including Taiwan is of wide international concern because it is easy for the viruses to spread from Asia to other countries [9]. Recently, 44 epidemic influenza A strains isolated during 2004–2008 in Taiwan were analyzed to provide a profile of protein variability to better understand viral antigenic evolution. The tree topology revealed the NP and NS genes could each be segregated into two groups similar to those for the polymerase genes. In addition, new genetic variants occurred during the non-epidemic period and become the dominant strain after one or two seasons [10]. The functional domains of NP have been mapped in the primary structure of the molecule [11]. Several studies reveled that the NP is a target of cytotoxic T lymphocytes (CTL) and the epitopes recognized by CTL in the NP molecule [12], [13],[14],[15],[16],[17],[18]. However, the human influenza evolution and dynamics in Taiwan have not been well studied, and there is a strong need for such an analysis. The overall goal of this study is to investigate the evolution of the viruses circulating in Taiwan and to understand evolutionary pathway of NP genes for human influenza viruses. We reported 131 representative NP genes from Taiwan human isolates during 1979 to 2009 and reference sequences retrieved from public databases, from which we analyze the evolutionary variability of nucleoprotein of influenza A virus and population dynamics.

## Results

### Phylogenetic analysis

**Figure 1:**
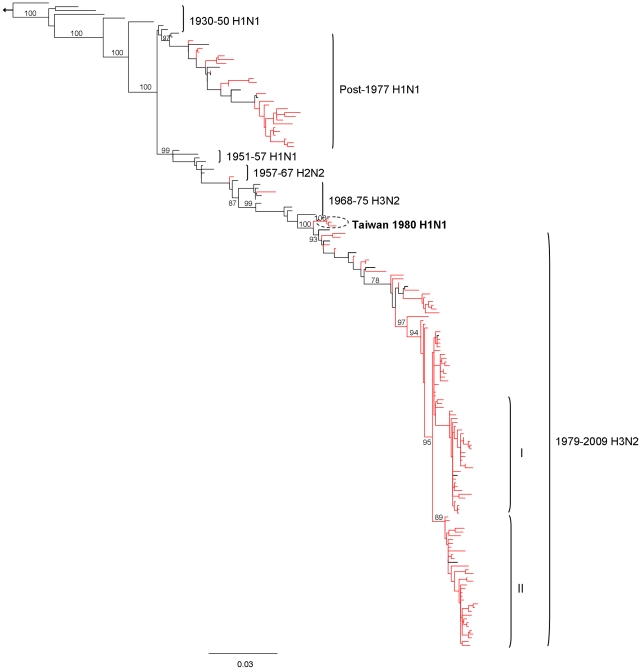
Phylogenetic ML tree of influenza A virus NP genes. Nucleotide tree rooted to A/Brevig Mission/1/1918. The arrow indicates the direction of the A/Brevig Mission/1/1918 NP from the root node. The dash-dot line circle denotes 3 1980 H1N1 viruses which grouped into the H3 lineage revealing the reassortment of internal gene segments of influenza A virus in Taiwan. The Taiwan isolates were coloured in red.

Analysis of the nucleotide sequences of the NP gene (positions 1–1464), in comparison with the 1918 H1N1 pandemic virus, the similarity for H1N1 and H3N2 were 90.6% ∼92.9% and 86.8% ∼90.2% respectively. However, the phylogenetic trees showed the H3 isolates were genetically divided into two clades (I and II) after the year 2004 (Figure1). Clade I comprised of the A/Wisconsin/67/2005 strain and related isolates from 2004 to 2007, and was characterized by two amino acids changes (Y52H and V280A) distinguishing it from clade II, which included the A/California/7/2004 strain and genetically similar isolates from 2004 to 2009. To make sure the observation of two clades was not artificially created by sample selection, Clade I and Clade II sequences were used to blast the NCBI GenBank. The new data set including all related sequences available (95% sequences similarity) were then used to build a maximum likelihood (ML) tree with bootstrapping (1000 replicates) analysis. The results showed other GenBank existing strains also genetically divided into these two clades. The amino acid sequences of the NP gene of H1 and H3 viruses differed from one another by 15 amino acids (Table 1).

**Table 1:** Amino acid differences of NP genes from influenza A virus H1 and H3 subtypes.

Position	191	217	286	309	334	343	344	353	375	408	423	446	450	452	459
H1	L	I	A	T	N	V	S	S	V	I/T	T	K	S	R	Q
H3	M	S	S	N	H	L	L	I/L	G/E	V	S	R	G	K	R

Additional 6 NP gene of the 2009 pandemic H1N1 (pdm H1N1) viruses were also analyzed (Figure S1). The selected viruses were chosen to be representative of all available sequences in Taiwan. Phylogenetic result of these pdm H1N1 isolates showed that these lineages are close to human H3N2 virus, but separated from all other isolates by a very long branch length with 100% bootstrap support. The monophyletic relationships between pdm H1N1 and seasonal H3N2 suggests either an increased rate of evolution or a long period during which the ancestors of the present epidemic went unsampled rather than separate interspecies transmission events from swine.

### Phylodynamic analysis

**Figure 2:**
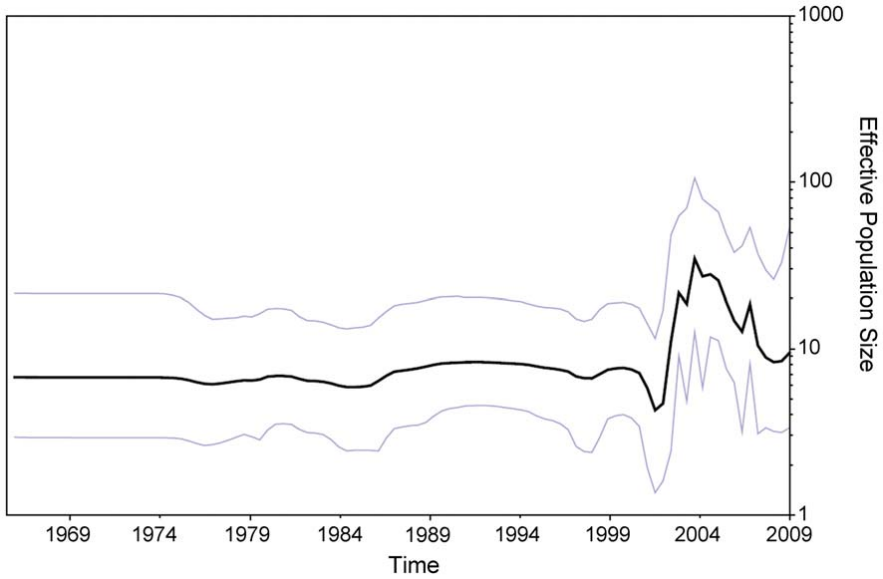
Bayesian skyline plot derived from an alignment of influenza nucleoprotein sequences in Taiwan. The x axis is in units of year before 2009, and the y axis represents the virus effective population size (i.e. the number of genomes effectively contributing to the next generation). The thick solid black line is the median estimate, and the pale blue lines show the upper and lower bounds of the 95% HDP interval.

BSP models were used to estimate the change of epidemic history and evolutionary dynamics of influenza A virus infection over time [8], [19], uncertainty in the estimated parameters was evaluated using 95% Highest Probability Density (HPD) intervals. The demographic inference using the BSP model is shown in Figure 2, the effective number of infections underwent a period of smooth and steady from 1998 continuously through 2001, followed by a more recent significant rise in the rate of spread. Noticeably, a sharp but transient increase in relative genetic diversity was observed from 2002, this corresponds to the period when H3N2 epidemic and reassortment event (acquired the internal gene segments from previous year) occurred in Taiwan and perhaps reflecting the occurrence of H1N1 and H3N2 co-circulations. The sharp peak in 2006–2007 was preceded by a decline in the effective population indicate of a purifying selection against the previous dominating strains. The BSP for the NP gene showed peaks for the effective population that coincided with the epidemic peaks observed in influenza surveillance system in Taiwan (Figure S2).

### Genetic variation and evolutionary rates

To elucidate the potential adaptive mutation of the recent isolates, we compared the human NPs with avian and swine NPs at the amino acid level. There are 14 positions that separate human viruses from avian and swine, as shown in the first column of Table 2. Such observation has been reported before as to be distinct or nearly distinct between human and avian viruses [20].Moreover, our data revealed that 3 of these 14 human positions (position 16, 283, and 313) have been conserved since 1918.

**Table 2:** Amino acid differences of NP genes from human, avian and swine isolates.

Human specific	Position	Avian	Swine	1918	1979–2009 H1/H3	2009 pdmH1
•	16*	G	G	D	D/D	G
	33	V	I	I	I/I	I
•	61	I	I	I	L/L	I
	98	R	R	R	K/RK	R
•	100	R	I	I	V/V	I
•	109	I	I	I	V/V	I
	136	L	M	M	I/MI	I
•	214	R	R	R	K/K	R
•	283*	L	L	P	P/P	L
	289	Y	H	Y	Y/Y	H
•	293	R	R	R	K/K	R
	305	R	R/K	R	K/K	K
•	313*	F	F	Y	Y/Y	V/I
•	372	E	E	E	D/D	E
•	375	D	D	D	V/EG	D
•	422	R	R	R	K/K	R
•	423	A	A	A	T/S	A
•	442	T	T	T	A/A	T
•	455	D	D	D	E/E	D

*Amino acids of NP at position 16, 283, and 313 were conserved for human viruses isolated since 1918.

Phylogenetic analysis of 171 sequences of influenza A virus showed that they segregated into two significant clades corresponding to the subtypes post-1977 H1 and H3. The branching pattern was supported both by maximum likelihood (bootstrap value ≥78%) and Bayesian analyses (posterior probability ≥0.82). The Bayes factors indicated that the relaxed clock did not yield significantly better results than the strict clock. The lower 95% HPD limit of the coefficient of variation and the evolutionary rate standard deviation estimates were small, indicating that the evolutionary rates varied only slightly among the branches of the tree. The evolutionary rates of the 1^st^+2^nd^ codon position (mean relative substitution rate 0.347, 95% HPD: 0.319–0.373) was not significantly different (two tails T-test p<0.01) from that observed at the 3^rd^ codon position (mean relative substitution rate 0.346, 95% HPD: 0.318–0.373) (Table 3).

**Table 3:** Mean relative evolutionary rates for codon positions in NP genes of influenza A virus.

	Mean relative substitution rate	SE of mean
1^st^ + 2^nd^ codon position (95% HPD)	0.347 (0.319–0.373)	1.414E-4
3^rd^ codon position (95% HPD)	0.346 (0.318–0.373)	1.471E-4

SE: standard error.

### Selection pressures on NP gene

The selection pressures acting on the NP gene of human influenza A viruses were estimated on the basis of the d_N_/d_S_ ratio to have a mean value of 0.105 with no significant difference between the H1 and H3 subtypes (H1∶0.091, H3∶0.119), thus suggesting the absence of any overall selection pressure on the portion of the protein analyzed. Moreover, the site-by-site analysis of selection pressure revealed 3 positively selected sites (position 197, 450, and 472) (Table 4) and 139 negatively selected sites out of 488 (28.5% at a 90% level of significance). This observation is different from previous report (1 positively selected and 62 negatively selected sites) [21]. However, 2 out of the 3 positively selected sites (position 197 and 472) were in the groups of functional amino acid sites involved in T-cell epitopes (TCEs) [22], [23]. These results suggest positively selected sites may be useful for identifying the epitopes involved in the protective immunity. Amino acid mutations in many of TCEs are expected to be advantageous, and generation of escape mutants in nucleoprotein has actually been reported [Boon et al, 2002].

**Table 4:** Positively selected sites in the NP protein of influenza A viruses at 90% significance level.

Site	p value SLAC	d_N_-d_S_
197	0.078	4.937
450	0.029	7.228
472	0.087	4.038

d_N_: non-synonymous sites; d_S_: synonymous sites.

## Discussion

To date, molecular evolution and phylogenetic analyses of influenza A viruses spanning successive seasons has not been reported for viruses circulating in Taiwan. The changing patterns of genetic diversity in viral isolates provide insight into the seasonal dynamics of influenza A and reveal evolutionary interactions between subtypes. Several studies have implicated the NP protein as a determinant of host range for influenza virus [24], [25], [26]. This suggests that the NP protein may be involved in the evolution and maintenance of distinct gene pools for influenza A viruses. NP has been proposed to be the gene responsible for keeping the two large, reservoirs of influenza A viruses in humans and water birds evolving separately [27], [28]. It is well-known that 1977 H1N1 is very likely a laboratory escape from the old H1N1 virus that disappeared after 1957 H2N2 pandemic [29], [30]. Therefore, 1977 H1N1 shared almost same genomic sequences as those of 1950s H1N1 sequences. This unnatural event, creating a phenomenon of “frozen evolution” between pre-1957 and post-1977 H1N1 viruses and will cause a large clock distortion in dating analyses. We use all the viruses including pre-1957 H1N1, post-1977H1N1, H2N2, and H3N2 exiting in NCBI GenBank together with the influenza A viruses circulating in Taiwan during 1979–2009 seasons to demonstrate the phylogenetic analyses.

Preliminary analysis of these NP genes did not reveal a genotypic difference between the strains from Taiwan and other countries (similarity 97.6–99.3% for H1N1 and 96.2–99.9% for H3N2). Phylogenetic analysis demonstrates that at least 2 clusters of H3N2 viruses cocirculated after 2004, but there was no temporal link between the groups because they were equally distributed throughout the study period. Furthermore, our previous report has revealed the co-circulation of multiple distinct groups and frequent intra-subtype reassortment events among them and such evolution plays an important role in antigenic drift [10]. Importantly, it should be noted that in 1980, 3 H1N1 viruses (A/Taiwan/509/1980, A/Taiwan/777/1980, and A/Taiwan/783/1980) grouped into the H3 lineage. These genetic variants represent the first detection of reassortment of the internal segments of influenza A viruses in Taiwan, but the reassortants did not persist after 1980. Moreover, phylogenetic analysis from our other project shows that not only the NP gene but also the polymerase genes (PB1, PB2, and PA) of these 3 H1N1 viruses were grouped into the H3 lineage. This provides evidence that genetic variation in influenza A H1N1 viruses is not restricted to mutation but also more likely undergo internal gene reassortment of which may have occurred sometime in 1980 or earlier. Dating the time of emergence of the 1980 H1 reassortants estimated their most recent common ancestor (MRCA) at 1950 (95% HPD: 1922–1981) for the NP gene. This result supports the hypothesis that the re-emergent H1N1/1977 virus was directly derived from those H1N1 viruses circulating in the 1950s [30]. The potential genetic parents for these 1980 H1 reassortants have not been detected, probably because little or no surveillance was conducted in Taiwan prior to the commencement of the monitoring of influenza activity by the Center for Diseases Control, Taiwan in 2000. Gene constellations of virus isolates from 1980 and later provided additional evidence of genetic exchange among co-circulating viruses.

To understand the evolutionary variability of nucleoprotein of influenza A virus and population dynamics, we used a flexible demographic model to examine the seasonal changes in genetic diversity of NP gene over the sampling time span [8]. This approach, based on the coalescent framework [31], allows the overall genetic diversity of a population to be estimated from a small sample of genetic sequences [32]. Thus an estimate of the change in genetic diversity over time can be obtained for a given genealogy. For these analyses, a nucleotide-substitution model was used that accommodates the different rates of and constraints upon evolution at different codon positions [33]. This allowed estimation of the overall rate of molecular evolution for each gene and the relative rate of the first and second codon positions compared with the third. We showed that these techniques confirm the epidemic behavior of the influenza A viruses. The first rise in the BSP was seen before 1999 (Figure 2), the time before the monitoring system of influenza activity has been coordinated by the Center for Diseases Control, Taiwan. Drastic and significant rise in 2002 and the estimated effective number was the highest in 2004 this coincide with phylogenetic analysis showing two distinct variants circulating in 2004. The BSP is in agreement with known epidemiological data for the past decade in Taiwan. Furthermore, comparing the BSP with epidemiological observations inform the influenza surveillance system in Taiwan, the BSP obtained from the NP gene showed a pattern that was in agreement with data on the H3N2 epidemics. In particular the 2002–2003, 2003–2005, and 2006–2007 epidemics, that were caused by the active circulation of H3N2, were reflected in the BSP. Therefore, our data suggest that the increase in the infected population was due to the H3N2 virus. It is possible that the relative fewer sequence available prior to 1999 in our dataset could have biased the estimate of the effective number of infections for this period and that the recent rise during 2002–2005, inferred in the BSP, could be a consequence of denser sampling after monitoring influenza activity started in 2000 (Table S1). Based on coalescent theory, however, a small random sample (≥8) of individuals from a given population can be used to construct phylogenetic relationships relating major viral lineages within the entire population [31], [34], and to determine the demographic history of pathogen's spread (i.e. the change in the number of effective infections over time, also referred to as a *skyline plot*) [35], [36]. In other words, as few as 8 sequences from epidemiologically unlinked subjects are usually sufficient to infer reliable molecular epidemiology parameters [34], [35], [37], Moreover, phylodynamic analysis of HA gene collected in Taiwan is proceeding by our other project, and the preliminary results (Figure S3) corroborate our finding here which reflect relative genetic diversity observed in recent years and corresponds to the period when H3N2 epidemic and reassortment event occurred in Taiwan. Taking together, two major changes were observed, epidemics arisen around 1999 were not able to detected and the growth phase after 2002 is preceded by an effective number of infections, which was not observed in previous coalescent analysis.

The NP gene has been reported to contain the highest number of species-associated amino acids suggesting that NP might serve as a molecular focus for differentiation between human and avian influenza A viruses [20]. A comparison of amino acid sequences showed that there are 14 human amino acids difference between avian and swine viruses, this is in agreement with the Chen et al (2006) analysis. Positions 375 and 423 were identified for the first time. Moreover, positions 16, 283, and 313 were observed to have been conserved since 1918. These three genomic signatures could be important markers of classical human influenza viruses. The emergence of the 2009 H1N1 virus pandemic (pdm H1N1) was the result of genetic reassortment [38], [39]. We also compared the NP gene of pdm H1N1 with the 14 human specific amino acid positions, except in position 313, the pdm H1N1 and swine isolates is nearly same but different from human. The pdm H1N1 retained the genetic host range markers suggesting that it is still able to further adapt to human viand that pig infection may function as a mixing vessel for the generation of new human influenza A viruses. Prospectively monitoring changes at these key positions in NP may be useful in identifying precursors of new genetic variants capable of causing future influenza epidemics or pandemics. In addition, it is interesting that usually we would expect the 3^rd^ codon position to evolve faster, however, only 3 positively selected sites were confirmed by the estimate of site-by-site selection pressure under significant positive selection in our study. On the contrary, about 28.5% of the residues constituting the NP segment analyzed were under 90% significant level of negative pressure, this could explain why that the synonymous (3^rd^ codon position) and non-synonymous (1+2 codon position) rate were not different.

Evolutionary divergence in human influenza H1 and H3 subtypes is probably related to differences in immune protection as it is related to host population structure. The correlates of interspecies transmission of influenza A virus genes from other host reservoirs to the human population remains unclear. The genetic variations of the NP gene will be helpful for monitoring the viruses and preparing effective prevention and controls for potential influenza outbreaks.

## Materials and Methods

### Ethics Statement

This study has been approved by the Institutional Review Board of Centers for Disease Control, R.O.C. (Taiwan) (DOH96-DC2402). The consent was waived for this study as there was no personal information collected from subjects.

### Isolates

Influenza A virus strains circulating in Taiwan during 1979–2009 seasons were isolated from selected patients with influenza-like illness presenting to physicians in sentinel practices. According to the Communicable Disease Control Act, all suspected influenza severe complicated cases need to be reported and collected specimen to Centers for Disease Control, R.O.C. (Taiwan) through National Notifiable Disease Surveillance System (NNDSS). The biological materials in this study were used only for standard diagnostic procedures following physicians' prescriptions and were conducted in accordance with no specific sampling, no modification of the sampling protocol. Analysis of data was performed using an anonymous database. Following local regulations, the procedure did not require a specific consent from patients. In brief, 100 µL of specimen from viral transport medium were inoculated into Madin-Darby canine kidney (MDCK) (CCL-34; American Type Culture Collection, Rockville, Md.) cells and incubated for 7–10 days. A positive cytopathic effect was confirmed by indirect immunofluorescence assay (IFA, Chemicon, Inc. Temecula, CA). Supernatants from positive cultures were harvested and stored for antigenic and genetic analysis.

### Virus detection and sequencing

For genotyping, 200 µL cultured supernatant were used for viral RNA extraction. Viral RNA was extracted using either the QIAamp Viral RNA Mini Kit (Qiagen, Santa Clara, CA) or an automated NucliSens easyMAG instrument (bioMerieux, Marcy l'Etoile, France). RT-PCR reactions were performed using a Perkin Elmer 2700 thermocycler (Applied Biosystems, Forest City, CA). The PCR product was purified using QIAquick spin columns using the manufacturer's protocols (Qiagen, Valencia, CA). Amplicons were cycle sequenced by using BigDye 3.1 Terminator cycle sequencing reagents with reaction products resolved on an ABI Prism 3130XL DNA Analyzer (Applied Biosystems, Forest City, CA).

### Phylogenetic analysis

All sequences of NP gene were aligned and use an interactive and hierarchical multiple-logo visualization tool, Phylo-mlogo [40], base on amino acids composition for grouping, and then the gene dataset for analysis were selected from each group. The number of sequences included in the final data set was 171, a dataset of 131 representative NP genes from Taiwan human isolates during 1979 to 2009 and 40 reference sequences retrieved from public databases (NCBI Influenza Virus Resource (www.ncbi.nlm.nih.gov/genomes/FLU/FLU.html), and the Global Initiative on Sharing All Influenza Data (GISAID; platform.gisaid.org). These reference sequence were collected between 1918 and 2009 in different areas of the world, including sequences which has been recommended by WHO for vaccine strains in different influenza seasons and hemispheres.

Multiple sequence alignments were performed and nucleotide sequences data analyzed using MEGA 4 software [41]. The best-fitting nucleotide substitution model was estimated by means of a hierarchical likelihood ratio approach using jModeltest program [42], and an HKY model [43] with gamma-distributed rates among sites selected. The likelihood estimates were made using PAUP* software version 4.0 [44]. Phylogeny reconstruction and evaluation were performed with the maximum likelihood (ML) and neighbor-joining (NJ) methods in the Phylip software package (version 3.66, University of Washington, Seattle, WA, USA) [45]. Empirical transition/transversion ratio and base frequency were estimated by the TREE-PUZZLE software (version 5.2) [46] and used to calculate evolutionary distances in jModeltest program [42].

### Phylodynamic analysis

The evolutionary rates, phylogenetic trees posterior distribution, population growth, and model parameters were estimated using Bayesian Markov Chain Monte Carlo (MCMC) method implemented in BEAST v1.5.4 package (http://beast.bio.ed.ac.uk) [47]. The posterior probability of each monophyletic clade was calculated to obtain statistical support for specific clades. In addition, as coalescent priors, we compared the constant and exponential growth parametric demographic models, and jModeltest program [42] was applied to the statistical selection of models of nucleotide substitution for the Bayesian analysis. In order to estimate the evolutionary dates and demographic history, we performed a Bayesian Skyline coalescent tree prior (BSP, non-parametric piecewise-constant model) [8] under the GTR + I + Γ nucleotide substitution model and using both strict and relaxed (with lognormal distribute rates) molecular clock model [48]. We use the best fit clock model to estimate a posterior distribution and then use as an empirical prior distribution in the coalescent analyses. The MCMC chain was run for 30 million generations, sampling every 30,000 steps. Proper mixing of the Markov Chain was assessed by calculating the effective sampling size (ESS) with Tracer software (http://tree.bio.ed.ac.uk/software/tracer/) after discarding the first 10% of the MCMC samples as “burn-in”. All estimated parameters showed ESS>250 indicating proper mixing. Uncertainty in the estimates was quantified by 95% highest posterior density (95% HPD) intervals and the tree with the maximum sum of posterior probabilities (maximum clade credibility) with a 10% burn-in was obtained from the posterior distribution with the LogCombiner v1.5.4 software. Tree topologies were finally annotated with Tree Annotator v1.5.4.

### Selection pressure

To determine the overall selection pressures for each gene, we estimated the mean numbers of nonsynonymous substitutions (d_N_) and synonymous substitutions (d_S_) per site (ratio d_N_/d_S_) using the single likelihood ancestor counting (SLAC) and fixed-effects likelihood (FEL) methods within the HYPHY package [49] through the Datamonkey web-based interface (http://www.datamonkey.org). The d_N_/d_S_ estimates were based on maximum likelihood (ML) trees under the HKY substitution model.

## Supporting Information

Figure S1 **Phylogenetic relationships of the NP gene segment of influenza A viruses from 1918 to 2009.** Forty global isolates and 131 Taiwan isolates from 1979 to 2009 used in this study and 6 pdmH1N1 Taiwan isolates (accession numbers:CY071403, CY071627, CY071635, CY073105, CY053474 CY047745), estimated using an ML method. The tree is rooted by the oldest isolate (A/Brevig Mission/1/1918).
(TIF)

Figures S2 **Weekly distribution of Taiwan influenza A isolates from 1999 to 2009.** Weekly distribution of Taiwan influenza A isolates based on cell culture results. The 2009 pandemic Influenza A (H1N1) virus is not included. Different color represent different subtypes: H1N1 in blue, H3N2 in red and yellow column shows subtyping were not performed.
(TIF)

Figures S3 **Bayesian skyline plot derived from an alignment of influenza hemagglutinin (HA) sequences in Taiwan.** The x-axis is in units of year before 2009, and the y-axis represents a measure of relative genetic diversity and reflects the number of effective infections established by the virus. The thick solid black line is the median estimate, and the pale blue lines show the upper and lower bounds of the 95% HDP interval.
(TIF)

Table S1 Accession number and distribution of the sequences over the time period of influenza A virus strains used in this study.
(DOC)

## References

[1] Rambaut A, Pybus OG, Nelson MI, Viboud C, Taubenberger JK (2008). The genomic and epidemiological dynamics of human influenza A virus.. Nature.

[2] Pybus OG, Rambaut A (2009). Evolutionary analysis of the dynamics of viral infectious disease.. Nat Rev Genet.

[3] Gammelin M, Altmuller A, Reinhardt U, Mandler J, Harley VR (1990). Phylogenetic analysis of nucleoproteins suggests that human influenza A viruses emerged from a 19th-century avian ancestor.. Mol Biol Evol.

[4] Gorman OT, Donis RO, Kawaoka Y, Webster RG (1990). Evolution of influenza A virus PB2 genes: implications for evolution of the ribonucleoprotein complex and origin of human influenza A virus.. J Virol.

[5] Kida H, Shortridge KF, Webster RG (1988). Origin of the hemagglutinin gene of H3N2 influenza viruses from pigs in China.. Virology.

[6] Lamb RA, Krug RM (1989). The genes and proteins of the influenza viruses.. The influenza viruses.

[7] Nakajima K, Desselberger U, Palese P (1978). Recent human influenza A (H1N1) viruses are closely related genetically to strains isolated in 1950.. Nature.

[8] Drummond AJ, Rambaut A, Shapiro B, Pybus OG (2005). Bayesian coalescent inference of past population dynamics from molecular sequences.. Mol Biol Evol.

[9] Russell CA, Jones TC, Barr IG, Cox NJ, Garten RJ (2008). The global circulation of seasonal influenza A (H3N2) viruses.. Science.

[10] Lin JH, Chiu SC, Cheng JC, Chang HW, Hsiao KL (2011). Molecular epidemiology and antigenic analyses of influenza A viruses H3N2 in Taiwan.. Clin Microbiol Infect.

[11] Portela A, Digard P (2002). The influenza virus nucleoprotein: a multifunctional RNA-binding protein pivotal to virus replication.. J Gen Virol.

[12] Fu TM, Friedman A, Ulmer JB, Liu MA, Donnelly JJ (1997). Protective cellular immunity: cytotoxic T-lymphocyte responses against dominant and recessive epitopes of influenza virus nucleoprotein induced by DNA immunization.. J Virol.

[13] Kreijtz JH, de Mutsert G, van Baalen CA, Fouchier RA, Osterhaus AD (2008). Cross-recognition of avian H5N1 influenza virus by human cytotoxic T-lymphocyte populations directed to human influenza A virus.. J Virol.

[14] Neumann G, Castrucci MR, Kawaoka Y (1997). Nuclear import and export of influenza virus nucleoprotein.. J Virol.

[15] Rimmelzwaan GF, Boon AC, Voeten JT, Berkhoff EG, Fouchier RA (2004). Sequence variation in the influenza A virus nucleoprotein associated with escape from cytotoxic T lymphocytes.. Virus Res.

[16] Voeten JT, Bestebroer TM, Nieuwkoop NJ, Fouchier RA, Osterhaus AD (2000). Antigenic drift in the influenza A virus (H3N2) nucleoprotein and escape from recognition by cytotoxic T lymphocytes.. J Virol.

[17] Townsend AR, Skehel JJ (1984). The influenza A virus nucleoprotein gene controls the induction of both subtype specific and cross-reactive cytotoxic T cells.. J Exp Med.

[18] Yewdell JW, Bennink JR, Smith GL, Moss B (1985). Influenza A virus nucleoprotein is a major target antigen for cross-reactive anti-influenza A virus cytotoxic T lymphocytes.. Proc Natl Acad Sci U S A.

[19] Minin VN, Bloomquist EW, Suchard MA (2008). Smooth skyride through a rough skyline: Bayesian coalescent-based inference of population dynamics.. Mol Biol Evol.

[20] Chen GW, Chang SC, Mok CK, Lo YL, Kung YN (2006). Genomic signatures of human versus avian influenza A viruses.. Emerg Infect Dis.

[21] Suzuki Y (2006). Natural selection on the influenza virus genome.. Mol Biol Evol.

[22] Andersen MH, Sondergaard I, Zeuthen J, Elliott T, Haurum JS (1999). An assay for peptide binding to HLA-Cw*0102.. Tissue Antigens.

[23] Gianfrani C, Oseroff C, Sidney J, Chesnut RW, Sette A (2000). Human memory CTL response specific for influenza A virus is broad and multispecific.. Hum Immunol.

[24] Scholtissek C, Burger H, Kistner O, Shortridge KF (1985). The nucleoprotein as a possible major factor in determining host specificity of influenza H3N2 viruses.. Virology.

[25] Reid AH, Fanning TG, Janczewski TA, Lourens RM, Taubenberger JK (2004). Novel origin of the 1918 pandemic influenza virus nucleoprotein gene.. J Virol.

[26] Varich NL, Sadykova GK, Prilipov AG, Kochergin-Nikitsky KS, Kushch AA (2011). Antibody-binding epitope differences in the nucleoprotein of avian and Mammalian influenza a viruses.. Viral Immunol.

[27] Hinshaw VS, Webster RG, Beare AS (1982). The natural history of influenza A viruses.. Basic and Applied Influenza Research.

[28] Kawaoka Y, Chambers TM, Sladen WL, Webster RG (1988). Is the gene pool of influenza viruses in shorebirds and gulls different from that in wild ducks?. Virology.

[29] Kilbourne ED (2006). Influenza pandemics of the 20th century.. Emerg Infect Dis.

[30] Smith GJ, Bahl J, Vijaykrishna D, Zhang J, Poon LL (2009). Dating the emergence of pandemic influenza viruses.. Proc Natl Acad Sci U S A.

[31] Kingman JFC (1982). The Coalescent Stoch. Proc Appl.

[32] Felsenstein J (2005). Using the quantitative genetic threshold model for inferences between and within species.. Philos Trans R Soc Lond B Biol Sci.

[33] Shapiro B, Rambaut A, Drummond AJ (2006). Choosing appropriate substitution models for the phylogenetic analysis of protein-coding sequences.. Mol Biol Evol.

[34] Felsenstein J (2006). Accuracy of coalescent likelihood estimates: do we need more sites, more sequences, or more loci?. Mol Biol Evol.

[35] Pybus OG, Rambaut A, Harvey PH (2000). An integrated framework for the inference of viral population history from reconstructed genealogies.. Genetics.

[36] Strimmer K, Pybus OG (2001). Exploring the demographic history of DNA sequences using the generalized skyline plot.. Mol Biol Evol.

[37] Lemey P, Salemi M, Vandamme AM (2009). The Phylogenetic Handbook: A Practical Approach to Phylogenetic Analysis and Hypothesis Testing..

[38] Garten RJ, Davis CT, Russell CA, Shu B, Lindstrom S (2009). Antigenic and genetic characteristics of swine-origin 2009 A(H1N1) influenza viruses circulating in humans.. Science.

[39] Smith GJ, Vijaykrishna D, Bahl J, Lycett SJ, Worobey M (2009). Origins and evolutionary genomics of the 2009 swine-origin H1N1 influenza A epidemic.. Nature.

[40] Shih AC, Lee DT, Peng CL, Wu YW (2007). Phylo-mLogo: an interactive and hierarchical multiple-logo visualization tool for alignment of many sequences.. BMC Bioinformatics.

[41] Tamura K, Dudley J, Nei M, Kumar S (2007). MEGA4: Molecular Evolutionary Genetics Analysis (MEGA) software version 4.0.. Mol Biol Evol.

[42] Posada D (2008). jModelTest: phylogenetic model averaging.. Mol Biol Evol.

[43] Hasegawa M, Kishino H, Yano T (1985). Dating of the human-ape splitting by a molecular clock of mitochondrial DNA.. J Mol Evol.

[44] Wilgenbusch JC, Swofford D (2003). Inferring evolutionary trees with PAUP*.. Curr Protoc Bioinformatics Chapter.

[45] Rodrigo AG, Felsenstein J, Crandall KA (1999). Coalescent approaches to HIV population genetics.. The evolution of HIV.

[46] Schmidt HA, Strimmer K, Vingron M, von Haeseler A (2002). TREE-PUZZLE: maximum likelihood phylogenetic analysis using quartets and parallel computing.. Bioinformatics.

[47] Drummond AJ, Rambaut A (2007). BEAST: Bayesian evolutionary analysis by sampling trees.. BMC Evol Biol.

[48] Drummond AJ, Ho SY, Phillips MJ, Rambaut A (2006). Relaxed phylogenetics and dating with confidence.. PLoS Biol.

[49] Pond SL, Frost SD, Muse SV (2005). HyPhy: hypothesis testing using phylogenies.. Bioinformatics.

