# Supplementary figures and images for: Phylodynamics and Molecular Evolution of Influenza A Virus Nucleoprotein Genes in Taiwan between 1979 and 2009
Source: PLoS One. 2011 Aug 12;6(8):e23454. doi: 10.1371/journal.pone.0023454 (PMC3155553; doi:10.1371/journal.pone.0023454)

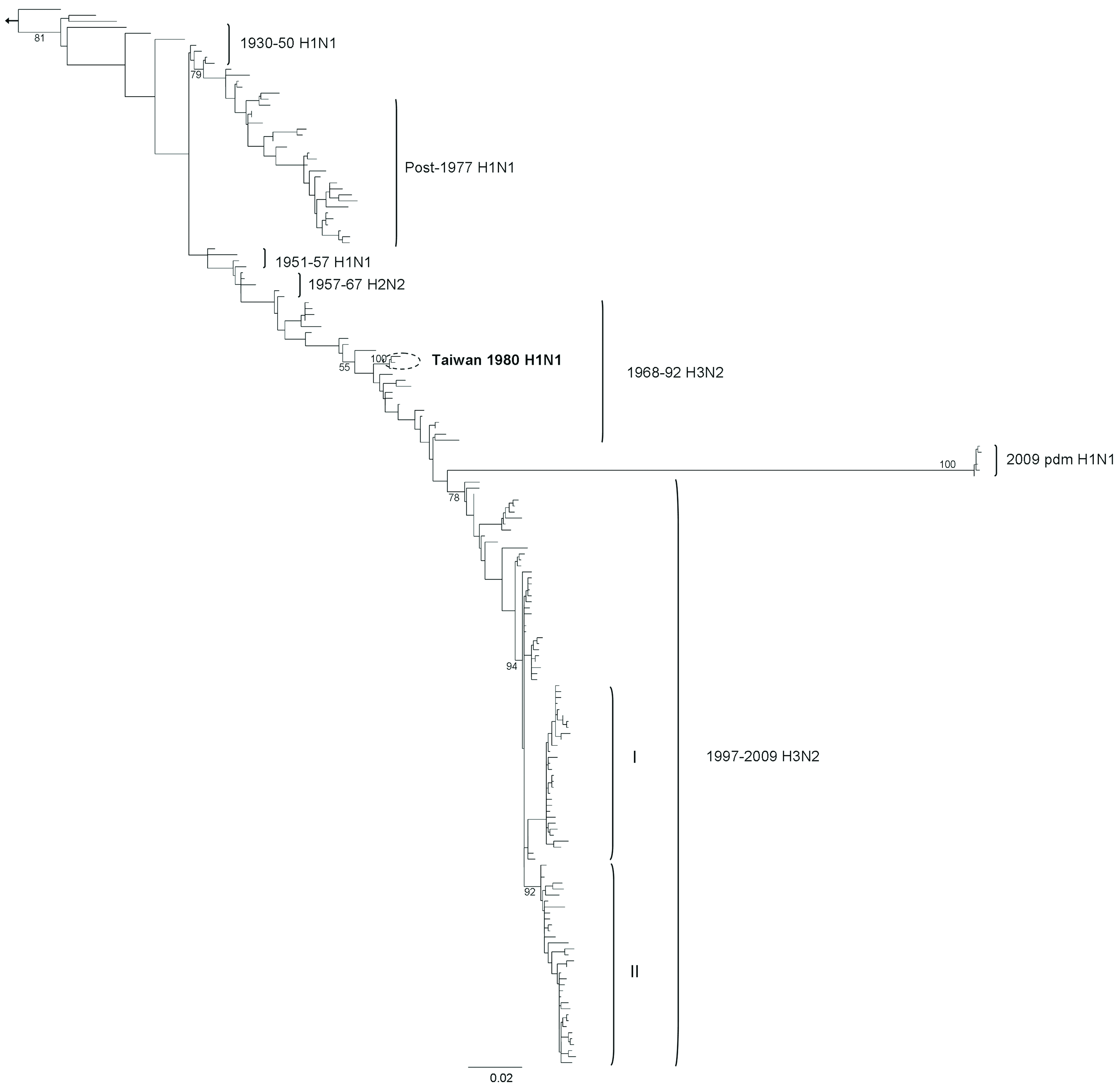

Supplement: Figure S1 — Phylogenetic relationships of the NP gene segment of influenza A viruses from 1918 to 2009. Forty global isolates and 131 Taiwan isolates from 1979 to 2009 used in this study and 6 pdmH1N1 Taiwan isolates (accession numbers:CY071403, CY071627, CY071635, CY073105, CY053474 CY047745), estimated using an ML method. The tree is rooted by the oldest isolate (A/Brevig Mission/1/1918). (TIF) [file pone.0023454.s001.tif]

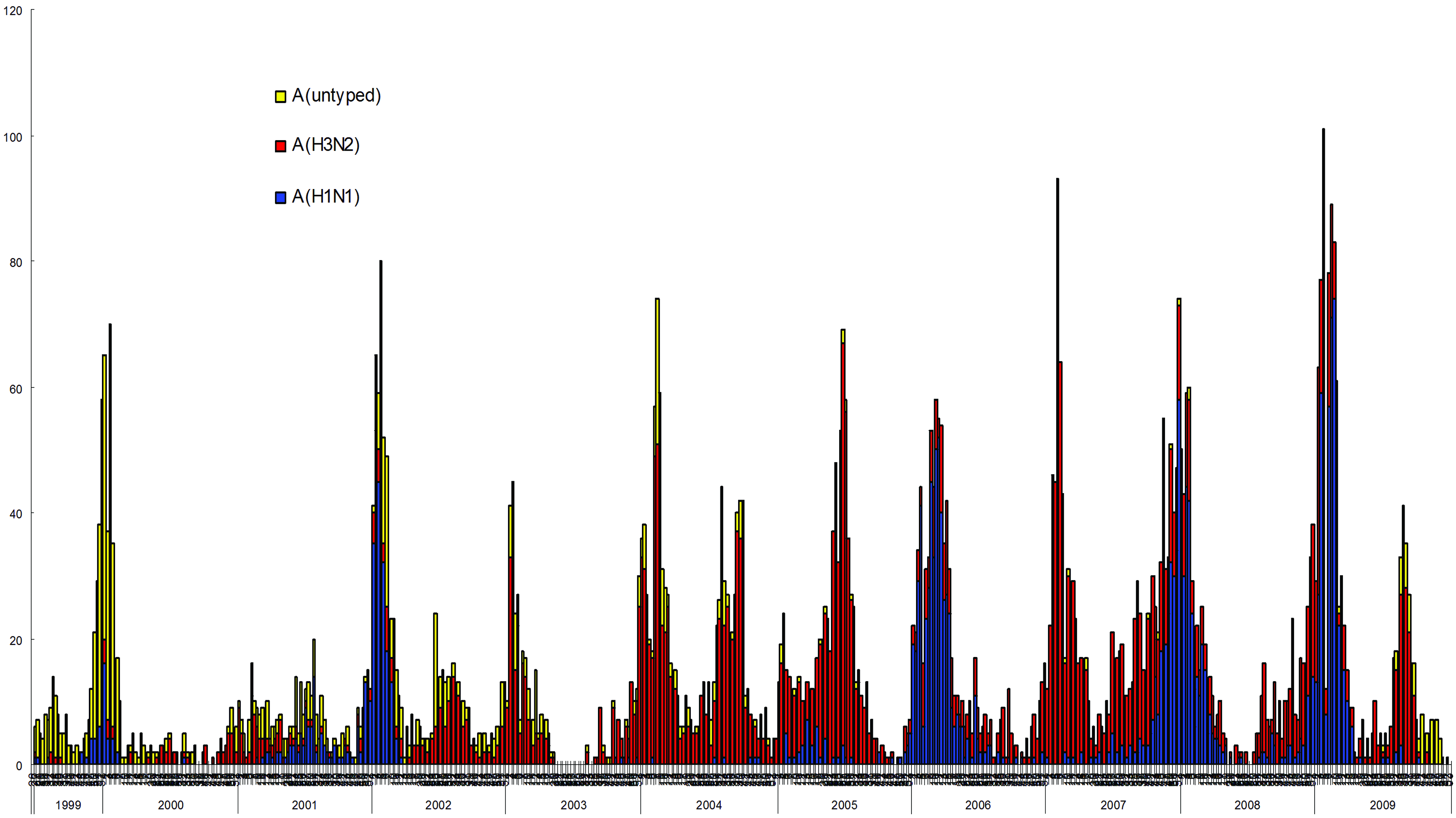

Supplement: Figures S2 — Weekly distribution of Taiwan influenza A isolates from 1999 to 2009. Weekly distribution of Taiwan influenza A isolates based on cell culture results. The 2009 pandemic Influenza A (H1N1) virus is not included. Different color represent different subtypes: H1N1 in blue, H3N2 in red and yellow column shows subtyping were not performed. (TIF) [file pone.0023454.s002.tif]

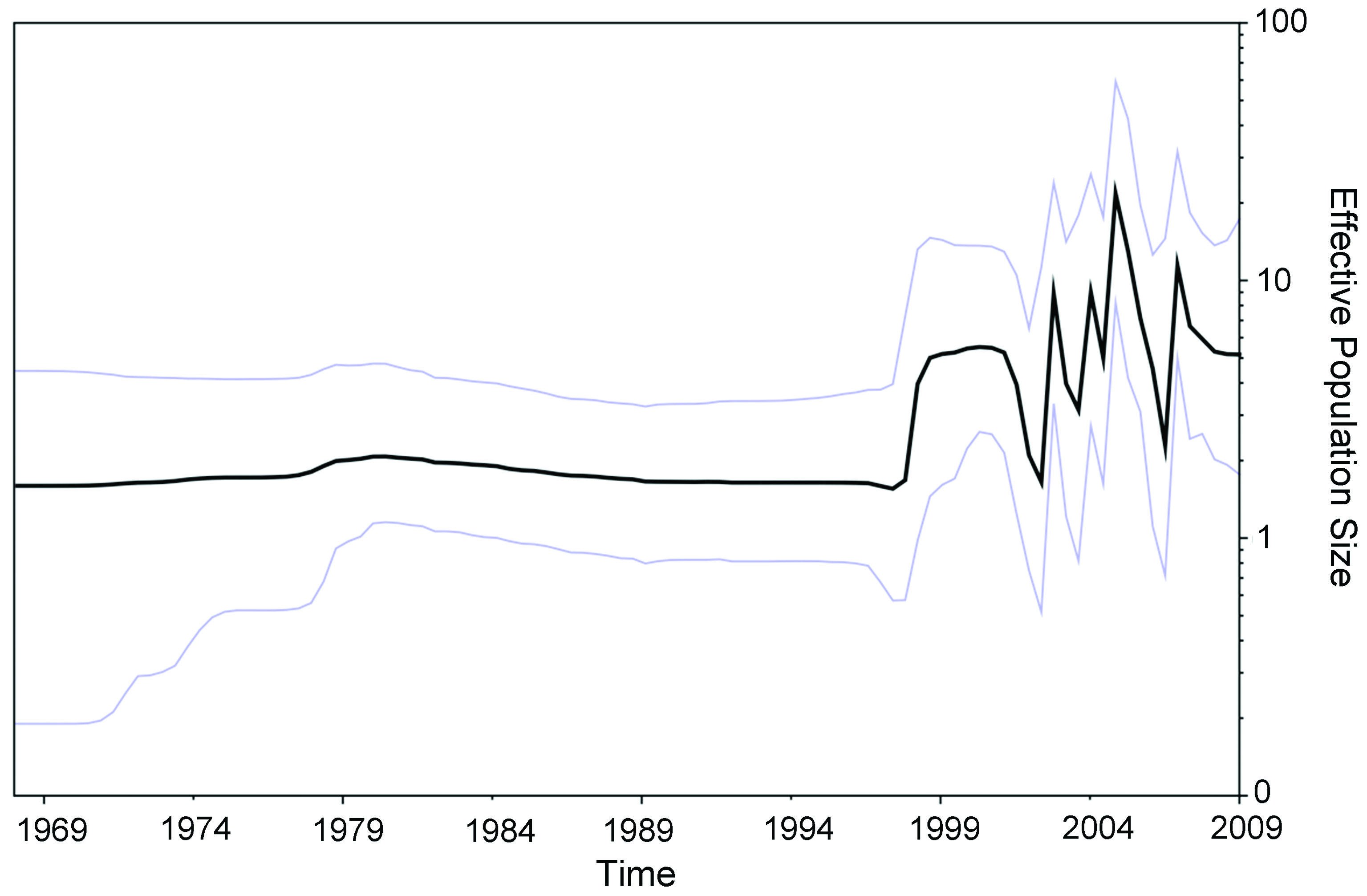

Supplement: Figures S3 — Bayesian skyline plot derived from an alignment of influenza hemagglutinin (HA) sequences in Taiwan. The x-axis is in units of year before 2009, and the y-axis represents a measure of relative genetic diversity and reflects the number of effective infections established by the virus. The thick solid black line is the median estimate, and the pale blue lines show the upper and lower bounds of the 95% HDP interval. (TIF) [file pone.0023454.s003.tif]
