# Supplementary material for: Phylodynamics and Molecular Evolution of Influenza A Virus Nucleoprotein Genes in Taiwan between 1979 and 2009
Source: PLoS One. 2011 Aug 12;6(8):e23454. doi: 10.1371/journal.pone.0023454 (PMC3155553; doi:10.1371/journal.pone.0023454)
Supplement: Table S1 — Accession number and distribution of the sequences over the time period of influenza A virus strains used in this study. (DOC) [file pone.0023454.s004.doc]

Table S1. Accession number and distribution of the sequences over the time period of influenza A virus strains used in this study.

| **Strain name** | **Accession number** | **Year** | **Subtype** |
| --- | --- | --- | --- |
| A/Brevig Mission/1/1918 | AY744935 | 1918 | H1N1 |
| A/WSN/1933 | CY034135 | 1933 | H1N1 |
| A/Alaska/1935 | CY019958 | 1935 | H1N1 |
| A/Bellamy/1942 | CY009279 | 1942 | H1N1 |
| A/Cameron/1946 | CY009599 | 1946 | H1N1 |
| A/Roma/1949 | CY019974 | 1949 | H1N1 |
| A/Fort Warren/1/1950 | D00601 | 1950 | H1N1 |
| A/Albany/1618/1951 | CY021904 | 1951 | H1N1 |
| A/Malaya/302/1954 | CY021056 | 1954 | H1N1 |
| A/England/19/1955 | M63751 | 1955 | H1N1 |
| A/Denver/1957 | CY008991 | 1957 | H1N1 |
| A/Loygang/4/1957 | M76604 | 1957 | H1N1 |
| A/USSR/90/1977 | CY010375 | 1977 | H1N1 |
| A/Brazil/11/1978 | CY020296 | 1978 | H1N1 |
| A/Taiwan/509/1980 | CY091968 | 1980 | H1N1 |
| A/Taiwan/777/1980 | CY091970 | 1980 | H1N1 |
| A/Taiwan/783/1980 | CY091971 | 1980 | H1N1 |
| A/Taiwan/927/1981 | CY091972 | 1981 | H1N1 |
| A/Taiwan/1020/1982 | CY091975 | 1982 | H1N1 |
| A/Chile/1/1983 | CY020440 | 1983 | H1N1 |
| A/Taiwan/1037/1984 | CY091976 | 1984 | H1N1 |
| A/Taiwan/1073/1986 | CY091978 | 1986 | H1N1 |
| A/Taiwan/1/1986 | DQ508874 | 1986 | H1N1 |
| A/Singapore/6/1986 | CY020480 | 1986 | H1N1 |
| A/Taiwan/1092/1987 | CY091980 | 1987 | H1N1 |
| A/Taiwan/1103/1988 | CY091982 | 1988 | H1N1 |
| A/Taiwan/1107/1988 | CY091983 | 1988 | H1N1 |
| A/Taiwan/1132/1991 | CY091984 | 1991 | H1N1 |
| A/Beijing/262/1995 | CY033617 | 1995 | H1N1 |
| A/Taiwan/960100/1996 | CY091986 | 1996 | H1N1 |
| A/Taiwan/960123/1996 | CY091987 | 1996 | H1N1 |
| A/Taiwan/343/1999 | CY091991 | 1999 | H1N1 |
| A/New Caledonia/20/1999 | CY033625 | 1999 | H1N1 |
| A/Taiwan/2/2000 | CY091992 | 2000 | H1N1 |
| A/Taiwan/188/2001 | CY091996 | 2001 | H1N1 |
| A/Taiwan/379/2001 | CY091997 | 2001 | H1N1 |
| A/Taiwan/286/2004 | CY092018 | 2004 | H1N1 |
| A/Taiwan/284/2004 | CY092019 | 2004 | H1N1 |
| A/Taiwan/135/2005 | CY092048 | 2005 | H1N1 |
| A/Taiwan/533/2005 | CY092045 | 2005 | H1N1 |
| A/Taiwan/603/2005 | CY092046 | 2005 | H1N1 |
| A/Taiwan/162/2006 | CY092059 | 2006 | H1N1 |
| A/Taiwan/217/2007 | CY092061 | 2007 | H1N1 |
| A/Taiwan/546/2007 | CY092062 | 2007 | H1N1 |
| A/Taiwan/547/2007 | CY092063 | 2007 | H1N1 |
| A/Taiwan/13/2008 | CY092079 | 2008 | H1N1 |
| A/Taiwan/6/2009 | CY092090 | 2009 | H1N1 |
| A/Guiyang/1/1957 | CY032272 | 1957 | H2N2 |
| A/Krasnodar/101/1959 | CY032256 | 1959 | H2N2 |
| A/Taiwan/1/1962 | AY210077 | 1962 | H2N2 |
| A/North Carolina/1/1963 | CY045791 | 1963 | H2N2 |
| A/Moscow/1019/1965 | CY031601 | 1965 | H2N2 |
| A/Tokyo/3/1967 | AY210096 | 1967 | H2N2 |
| A/Hong Kong/1/1968 | AF348180 | 1968 | H3N2 |
| A/Northern Territory/60/1968 | CY011123 | 1968 | H3N2 |
| A/Alaska/1/1969 | AY210224 | 1969 | H3N2 |
| A/Albany/6/1970 | CY021120 | 1970 | H3N2 |
| A/Taiwan/3/1971 | AY210230 | 1971 | H3N2 |
| A/England/42/1972 | AY210238 | 1972 | H3N2 |
| A/Hong Kong/11/1973 | CY003531 | 1973 | H3N2 |
| A/Victoria/3/1975 | AF072545 | 1975 | H3N2 |
| A/Guangdong/38/1977 | L07359 | 1977 | H3N2 |
| A/Taiwan/10/1979 | CY091967 | 1979 | H3N2 |
| A/Bangkok/1/1979 | DQ508826 | 1979 | H3N2 |
| A/Taiwan/601/1980 | CY091969 | 1980 | H3N2 |
| A/Taiwan/929/1981 | CY091973 | 1981 | H3N2 |
| A/Taiwan/1021/1982 | CY091975 | 1982 | H3N2 |
| A/Memphis/2/1985 | L07362 | 1985 | H3N2 |
| A/Taiwan/1069/1985 | CY091977 | 1985 | H3N2 |
| A/Taiwan/1097/1987 | CY091981 | 1987 | H3N2 |
| A/Taiwan/1091/1987 | CY091979 | 1987 | H3N2 |
| A/Beijing/32/1992 | CY033609 | 1992 | H3N2 |
| A/Taiwan/1151/1992 | CY091985 | 1992 | H3N2 |
| A/Taiwan/1342/1997 | CY091990 | 1997 | H3N2 |
| A/Taiwan/1335/1997 | CY091988 | 1997 | H3N2 |
| A/Taiwan/1338/1997 | CY091989 | 1997 | H3N2 |
| A/Panama/2007/1999 | CY034103 | 1999 | H3N2 |
| A/Taiwan/431/2000 | CY091994 | 2000 | H3N2 |
| A/Taiwan/235/2000 | CY091993 | 2000 | H3N2 |
| A/Taiwan/384/2001 | CY091998 | 2001 | H3N2 |
| A/Taiwan/55/2001 | CY091999 | 2001 | H3N2 |
| A/Taiwan/124/2001 | CY091995 | 2001 | H3N2 |
| A/Taiwan/478/2002 | CY092002 | 2002 | H3N2 |
| A/Taiwan/92/2002 | CY092003 | 2002 | H3N2 |
| A/Taiwan/231/2002 | CY092000 | 2002 | H3N2 |
| A/Taiwan/299/2002 | CY092001 | 2002 | H3N2 |
| A/Wyoming/3/2003 | EU268228 | 2003 | H3N2 |
| A/Taiwan/12/2003 | CY092011 | 2003 | H3N2 |
| A/Taiwan/25/2003 | CY092012 | 2003 | H3N2 |
| A/Taiwan/27/2003 | CY092014 | 2003 | H3N2 |
| A/Taiwan/31/2003 | CY092013 | 2003 | H3N2 |
| A/Taiwan/47/2003 | CY092015 | 2003 | H3N2 |
| A/Taiwan/62/2003 | CY092016 | 2003 | H3N2 |
| A/Taiwan/73/2003 | CY092017 | 2003 | H3N2 |
| A/Taiwan/78/2003 | CY092004 | 2003 | H3N2 |
| A/Taiwan/88/2003 | CY092005 | 2003 | H3N2 |
| A/Taiwan/89/2003 | CY092006 | 2003 | H3N2 |
| A/Taiwan/139/2003 | CY092007 | 2003 | H3N2 |
| A/Taiwan/142/2003 | CY092008 | 2003 | H3N2 |
| A/Taiwan/145/2003 | CY092010 | 2003 | H3N2 |
| A/Taiwan/146/2003 | CY092009 | 2003 | H3N2 |
| A/California/7/2004 | CY047417 | 2004 | H3N2 |
| A/Taiwan/925/2004 | CY092025 | 2004 | H3N2 |
| A/Taiwan/926/2004 | CY092026 | 2004 | H3N2 |
| A/Taiwan/927/2004 | CY092028 | 2004 | H3N2 |
| A/Taiwan/931/2004 | CY092021 | 2004 | H3N2 |
| A/Taiwan/933/2004 | CY092022 | 2004 | H3N2 |
| A/Taiwan/935/2004 | CY092023 | 2004 | H3N2 |
| A/Taiwan/693/2004 | CY092024 | 2004 | H3N2 |
| A/Taiwan/983/2004 | CY092027 | 2004 | H3N2 |
| A/Taiwan/393/2004 | CY092031 | 2004 | H3N2 |
| A/Taiwan/1103/2004 | CY092029 | 2004 | H3N2 |
| A/Taiwan/371/2004 | CY092030 | 2004 | H3N2 |
| A/Taiwan/399/2004 | CY092032 | 2004 | H3N2 |
| A/Taiwan/586/2004 | CY092033 | 2004 | H3N2 |
| A/Taiwan/21/2004 | CY092020 | 2004 | H3N2 |
| A/Taiwan/615/2004 | CY092034 | 2004 | H3N2 |
| A/Taiwan/627/2004 | CY092035 | 2004 | H3N2 |
| A/Taiwan/670/2004 | CY092037 | 2004 | H3N2 |
| A/Taiwan/330/2004 | FJ805610 | 2004 | H3N2 |
| A/Taiwan/269/2004 | CY092036 | 2004 | H3N2 |
| A/Wisconsin/67/2005 | CY034119 | 2005 | H3N2 |
| A/Taiwan/512/2005 | CY092038 | 2005 | H3N2 |
| A/Taiwan/80/2005 | CY092039 | 2005 | H3N2 |
| A/Taiwan/141/2005 | CY092040 | 2005 | H3N2 |
| A/Taiwan/156/2005 | FJ805615 | 2005 | H3N2 |
| A/Taiwan/280/2005 | CY092041 | 2005 | H3N2 |
| A/Taiwan/468/2005 | CY092042 | 2005 | H3N2 |
| A/Taiwan/17/2005 | CY092043 | 2005 | H3N2 |
| A/Taiwan/36/2005 | CY092044 | 2005 | H3N2 |
| A/Taiwan/108/2005 | CY092047 | 2005 | H3N2 |
| A/Taiwan/87/2006 | CY092053 | 2006 | H3N2 |
| A/Taiwan/717/2006 | CY092049 | 2006 | H3N2 |
| A/Taiwan/718/2006 | CY092050 | 2006 | H3N2 |
| A/Taiwan/754/2006 | FJ805626 | 2006 | H3N2 |
| A/Taiwan/100/2006 | CY092054 | 2006 | H3N2 |
| A/Taiwan/776/2006 | CY092051 | 2006 | H3N2 |
| A/Taiwan/788/2006 | CY092052 | 2006 | H3N2 |
| A/Taiwan/83/2006 | CY092056 | 2006 | H3N2 |
| A/Taiwan/98/2006 | CY092057 | 2006 | H3N2 |
| A/Taiwan/315/2006 | CY092060 | 2006 | H3N2 |
| A/Taiwan/586/2006 | CY092055 | 2006 | H3N2 |
| A/Taiwan/799/2006 | FJ805629 | 2006 | H3N2 |
| A/Taiwan/133/2006 | CY092058 | 2006 | H3N2 |
| A/Brisbane/10/2007 | CY035025 | 2007 | H3N2 |
| A/Taiwan/4/2007 | CY092071 | 2007 | H3N2 |
| A/Taiwan/34/2007 | CY092064 | 2007 | H3N2 |
| A/Taiwan/213/2007 | CY092065 | 2007 | H3N2 |
| A/Taiwan/279/2007 | CY092066 | 2007 | H3N2 |
| A/Taiwan/450/2007 | CY092067 | 2007 | H3N2 |
| A/Taiwan/511/2007 | CY092068 | 2007 | H3N2 |
| A/Taiwan/539/2007 | CY092069 | 2007 | H3N2 |
| A/Taiwan/541/2007 | CY092070 | 2007 | H3N2 |
| A/Taiwan/21/2007 | CY092073 | 2007 | H3N2 |
| A/Taiwan/33/2007 | FJ805633 | 2007 | H3N2 |
| A/Taiwan/52/2007 | CY092074 | 2007 | H3N2 |
| A/Taiwan/56/2007 | CY092075 | 2007 | H3N2 |
| A/Taiwan/58/2007 | CY092076 | 2007 | H3N2 |
| A/Taiwan/131/2007 | CY092077 | 2007 | H3N2 |
| A/Taiwan/303/2007 | CY092078 | 2007 | H3N2 |
| A/Taiwan/426/2007 | CY092072 | 2007 | H3N2 |
| A/Taiwan/3/2008 | CY092081 | 2008 | H3N2 |
| A/Taiwan/754/2009 | CY092082 | 2009 | H3N2 |
| A/Taiwan/191/2009 | CY092083 | 2009 | H3N2 |
| A/Taiwan/426/2009 | CY092085 | 2009 | H3N2 |
| A/Taiwan/556/2009 | CY092086 | 2009 | H3N2 |
| A/Taiwan/558/2009 | CY092087 | 2009 | H3N2 |
| A/Taiwan/631/2009 | CY092088 | 2009 | H3N2 |
| A/Taiwan/11/2009 | CY092089 | 2009 | H3N2 |
| A/Taiwan/188/2009 | CY092092 | 2009 | H3N2 |
| A/Taiwan/839/2009 | CY092091 | 2009 | H3N2 |
